# 506. Long-term Quality of Life Outcomes in Adult Burn Survivors: An Integrative Review

**DOI:** 10.1093/jbcr/irag033.165

**Published:** 2026-04-06

**Authors:** Sarah M Nesbitt

**Affiliations:** Kessler Burn Center, Webster, New York

## Abstract

**Introduction:**

Burn injuries affect nearly 400 000 individuals annually in the United States. While survival rates have improved with advances in acute care, survivors frequently experience persistent physical, psychological, and social challenges that compromise long-term quality of life.

**Methods:**

An integrative review was conducted following the Whittemore and Knafl framework. PubMed, CINAHL, and APA PsycINFO databases were searched for peer-reviewed studies on adult burn survivors in the United States, focusing on post-discharge quality of life. Seven studies met the inclusion criteria and were synthesized for analysis.

**Results:**

Burn survivors demonstrated sustained impairments across physical, emotional, and social domains. Chronic pain, mobility limitations, and fatigue contributed to reduced physical functioning. Emotional sequelae, including anxiety, depression, and PTSD, persisted years after injury, often worsened by dysfunctional coping strategies. Female survivors consistently reported a lower quality of life compared to males. Despite physical gains from reconstructive surgery, emotional recovery often lagged. Psychosocial and mental health support were inconsistently available, and stigma, functional limitations, and lack of support hindered social and occupational reintegration.

**Conclusions:**

Long-term burn recovery is complex, multidimensional, and inadequately addressed in current care models. The existing literature highlights the absence of standardized follow-up protocols and a lack of interventional studies focusing on quality of life outcomes.

**Applicability of Research to Practice:**

Advanced practice nurses are positioned to lead multidisciplinary follow-up care, screen for psychosocial distress, and coordinate interventions beyond acute care. Integrating structured, long-term follow-up into practice can improve burn survivors’ resilience, reintegration, and overall quality of life.

**Funding for the study:**

N/A.

